# Full-term pregnancy in a rudimentary horn with a live fetus: A case report

**DOI:** 10.1097/MD.0000000000021604

**Published:** 2020-08-21

**Authors:** Yu Zhang, Yingxin Pang, Xue Zhang, Zhe Zhao, Peishu Liu

**Affiliations:** Department of Obstetrics and Gynecology, Qilu Hospital, Cheeloo College of Medcine, Shandong University, Jinan, Shandong, China.

**Keywords:** case report, full-term pregnancy, rudimentary horn, unicornuate uterus

## Abstract

**Introduction:**

Rudimentary horns and unicornuate uteri, 2 types of Mullerian duct abnormalities, often lack obvious symptoms. Ultrasonography (US) and magnetic resonance imaging (MRI) are alternative examinations but have low accuracy. Full-term rudimentary horn pregnancies are rather rare but life-threatening.

**Patient concerns:**

A 30-year-old Chinese woman complained of lower abdominal pain one year after a full-term unicornate uterus pregnancy and a rudimentary horn pregnancy successively.

**Diagnosis:**

Uterine dysplasia (right rudimentary uterine horn and left unicornate uterus), hematometra and right fallopian tube effusion were diagnosed.

**Interventions:**

We performed laparoscopic hysterectomy (rudimentary horn), right salpingectomy, pelvic adhesion release and hysteroscopy.

**Outcomes:**

The patient has not complained of specific discomfort during the one-year follow-up so far.

**Conclusion:**

The reported case was a rare full-term rudimentary horn pregnancy. The degree of development of the rudimentary horn, such as the endometrial function, muscle layer thickness, and uterine shape and size, is closely related to pregnancy outcome. The rudimentary horn with a functional endometrium must be disposed of once it is definitely diagnosed. Pregnancy in the rudimentary horn with a weak muscular layer should be treated as soon as possible. Detailed and scientific prenatal examination is important.

## 1 Introduction

Mullerian duct abnormalities are rare, and the exact prevalence has not been well established because of the asymptomatic presentation. These abnormalities occur in approximately 1/200-1/600 of fertile women.^[1]^ Mullerian duct abnormalities may accompany congenital anomalies in the renal (with a prevalence of 30%–50%) and vertebral systems (with a prevalence of 29%).^[2]^ Unicornuate uteri and rudimentary horns are two types of Mullerian duct abnormalities. Pregnancy often occurs in the unicornuate uterus; however, it tends to end with abortion or premature and full-term delivery. Rudimentary horn pregnancy is rather rare, with an incidence of approximately 1/76,000 pregnancies.^[1]^ Ultrasonography (US) is the first-line diagnostic tool due to its convenient and radiationless nature. Magnetic resonance imaging (MRI) is superior in terms of multiplanar imaging and soft tissue characterization. However, the diagnostic accuracy of US and MRI is unsatisfactory. Only 8% of rudimentary horn pregnancies are diagnosed before symptoms appear.^[1]^ Pregnancy outcomes are commonly classified as abortion, preterm delivery, uterine rupture and, rarely, full-term pregnancy. The acknowledged principle of management remains undefined.

In this case, the patient experienced a full-term unicornuate uterus pregnancy and rudimentary horn pregnancy with healthy neonates and suffered from hysterectomy (rudimentary horn) one year after the second cesarean section due to menstrual blood retention. We aim to provide a typical case, summarize the associated clinical characteristics and therapies, and finally offer clinical experience for labor inspection and reasonable treatment.

## 2 Case presentation

A 30-year-old Chinese woman visited our hospital with a complaint of lower abdominal pain one year after a full-term unicornate uterus pregnancy and a rudimentary horn pregnancy successively. When the patient was 25 years old, she was suspected of having a uterine malformation according to prenatal examination but did not have a related family history. She received a regular physical examination, showing good results without abdominal pain, vaginal bleeding or any other discomfort. The pregnancy was terminated through a cesarean section at 38 weeks of gestation with a healthy neonate. During the operation, we found one case of a cervix uteri, right rudimentary horn and left unicornate uterus separately connected to the fallopian tube and ovary. This pregnancy occurred in the unicornate uterus. The fetus and placenta were delivered smoothly. The patient was expecting again four years later. She did not complain of any discomfort during gestation. Under close surveillance, she received a cesarean section at 38 weeks. A rudimentary horn pregnancy was diagnosed during the operation. The placenta was attached to the wall of the uterus and stripped by the obstetrician. Massaging the uterus was used to promote uterine contractions. The Apgar score of the newborn was 10. Menstruation accompanied by dysmenorrhea occurred 6 months after the second cesarean section with the same menstrual cycle and volume. The patient complained of gradually aggravated lower abdominal pain with low back pain one year after the second surgery.

**Figure 1:**
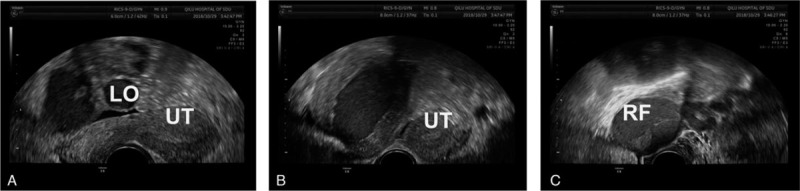
Ultrasound showing uterine dysplasia (right rudimentary uterine horn and left unicornate uterus) and hematometra of the reported case. A: left unicornate uterus and ovary (UT: uterus, LO: left ovary). B: hematometra in right rudimentary uterine horn. C: expansion of the right fallopian tube and ovary (RF: right fallopian tube).

**Figure 2:**
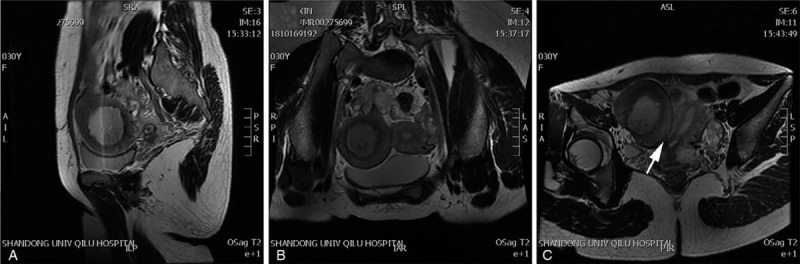
MRI showing uterine dysplasia (right rudimentary uterine horn and left unicornate uterus) and hematometra of the reported case. A: hematocele in rudimentary horn uterine cavity. B: right rudimentary horn uterine with hematocele and left unicornate uterus. C: no passage between rudimentary horn and cervix or unicornate uterus (arrow).

In terms of the gynecological examination, we palpated an anterior uterus with a normal size and a hard mass with a diameter of 7 cm, a smooth surface, poor activity and tenderness. Gynecological ultrasound (Fig. 1) found that the endometrium of the anterior uterus extended to the left side of the parametrium. There was a 7.3 × 6.3 × 5.8 cm low-echo signal similar to the muscular layer with a 5.2 × 3.9 cm fluid sonolucent area on the right side of the uterus. There was no connection between the low-echo signal and cervix. The ultrasound revealed the rudimentary uterine horn and uterine hemorrhage. MRI (Fig. 2) showed 2 uteri, of which the right uterus was larger and had no connection with the cervix. There was a cyst with a diameter of 4.6 cm in the right ovary region. A tube-like structure was attached to it. MRI indicated a uterine malformation (right rudimentary uterine horn, left unicornate uterus) and expansion of the right fallopian tube. Urinary ultrasound showed no abnormalities.

Combining the clinical manifestations and the gynecology and image examination results, we primarily diagnosed uterine dysplasia (right rudimentary uterine horn and left unicornate uterus), hematometra and right fallopian tube effusion.

**Figure 3:**
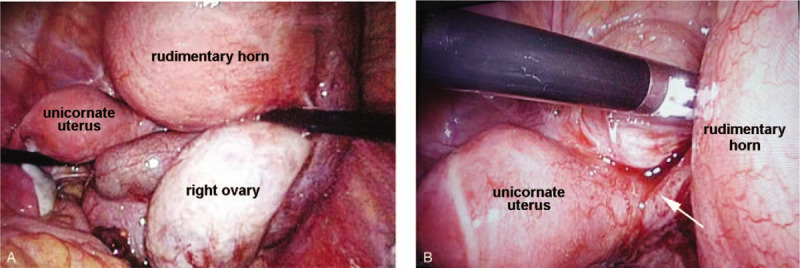
Pictures during the operation. A: left unicornate uterus and right rudimentary horn uterine with hematocele. B: fiber band between left unicornate uterus and right rudimentary horn(arrow).

**Figure 4:**
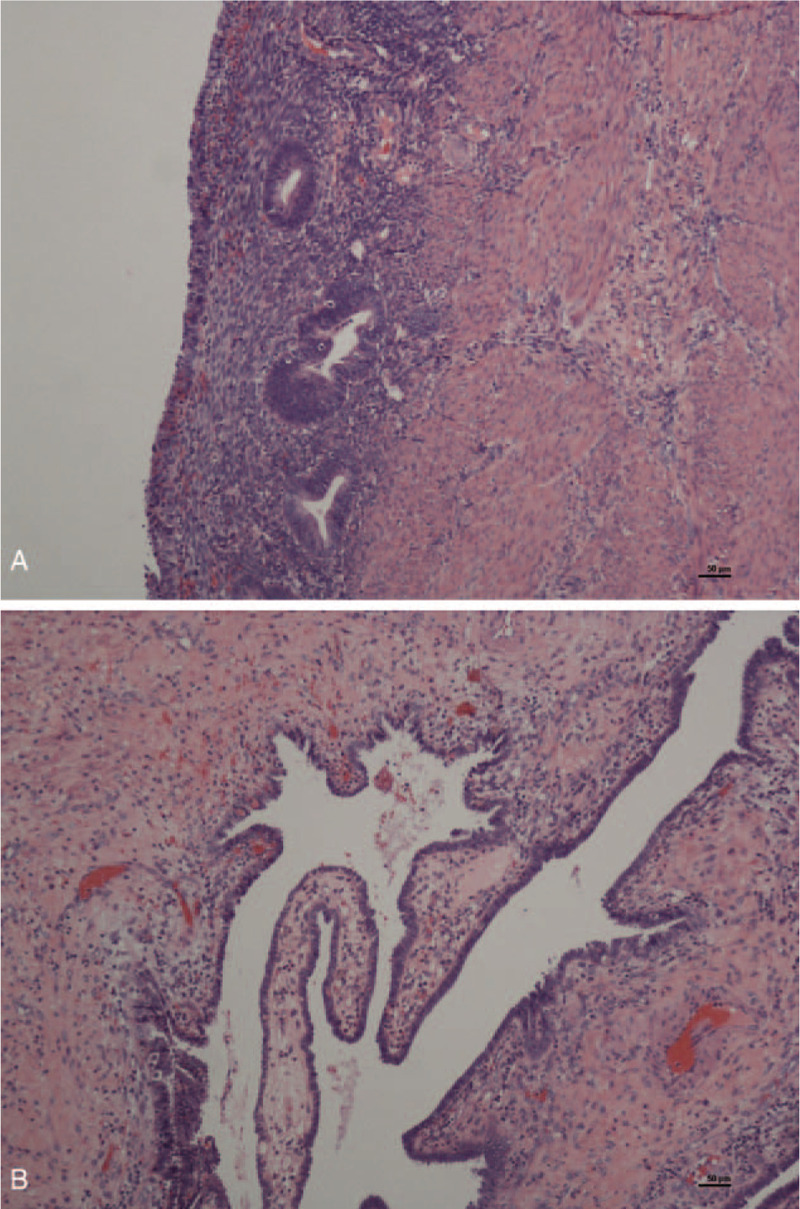
Postoperative pathology. A: Smooth muscle of rudimentary horn covered by endometrium is significantly hyperemic, edematous, and infiltrated by chronic inflammatory cells. B: Right fallopian tube is congestive, edematous, with chronic inflammation and cystic dilatation.

During laparoscopic exploration (Fig. 3), we observed two uterine bodies: the right rudimentary uterine horn was enlarged at three months of gestation, while the left unicornate uterus was of normal size. There was a fibrous band between the two uterine bodies. Two fallopian tubes and ovaries were linked with each uterus, of which the right tube and ovary were swollen. Under hysteroscopy, we only observed one cervix communicating with the left uterus and no obvious connection between the two uteri. Then, we performed laparoscopic hysterectomy (rudimentary horn) + right salpingectomy + pelvic adhesion release + hysteroscopy. The operation confirmed a unicornate uterus and rudimentary horn. Postoperative pathology (Fig. 4) suggested that the smooth muscle of the rudimentary horn covered by the endometrium was significantly hyperemic, edematous, and infiltrated by chronic inflammatory cells. The right fallopian tube was congestive and edematous, with chronic inflammation and cystic dilatation. Intrauterine adhesions after the second cesarean section closed the bridge between the two uterine bodies, which led to hematometra in the rudimentary horn. The patient has not complained of specific discomfort during the one-year follow-up so far.

## 3 Discussion

Rudimentary horn pregnancy is rarely seen. Early diagnosis of rudimentary horn pregnancy is of great importance because poor musculature can result in the life-threatening complication of uterine rupture. The rate of rupture may reach 80%.^[3]^ However, it is quite a challenge to diagnose rudimentary horn pregnancy, especially in the first trimester, due to the lack of typical symptoms. Rudimentary horn pregnancy usually accompanies fetal growth restriction, oligohydramnios, fetal demise, uterine rupture, preterm delivery or few full-term gestations, similar to the reported case. Only a few obstetrical risks can be reduced by a particular pregnancy follow-up and specific interventions. Ultrasound imaging is always the first choice, but the sensitivity is approximately 26% and might decrease in advanced pregnancies.^[4]^ Three-dimensional ultrasound (3D-ultrasound) has been proven to be more accurate.^[5]^ Compared with ultrasound, MRI has been considered the better noninvasive method of diagnosing Mullerian anomalies.^[6]^ MRI is superior in detecting a hypoplastic uterus, normal ovaries, a fibrous septum and soft tissue between the two uterine horns.^[2]^ Some invasive tests (hysterosalpingogram, hysteroscopy or laparoscopy) may contribute to the accurate pregestational diagnosis of uterine malformations. Therefore, comprehensive progestational and gestational check-ups are of great importance in the early diagnosis of uterine malformations.

Most pregnancies in a rudimentary horn end with torsion and rupture, and a few patients still have secondary abdominal pregnancies. The rudimentary horn musculature and hypertrophic ability are related to the time of rupture (mostly 5-35 weeks). A total of 61% and 6% of uterine ruptures occur in the second and third trimesters, respectively.^[7]^ Bhandary Amrithan^[8]^ and Ashma Rana^[9]^ reported two cases of abdominal pregnancies secondary to rudimentary horn pregnancy fractures. The formal gravida ended with 40 weeks of gestation through a cesarean section, and her female baby weighed 3 kg and had a good condition. The latter patient received an emergency cesarean section at 27^+5^ weeks gestation, and the baby weighing 650 g died on the third day.

There are still some full-term rudimentary horn pregnancies (Table 1). Chou et al^[10]^ and Chen Cheng et al^[1]^ both reported term pregnancies in a noncommunicating rudimentary horn of a unicornuate uterus with a healthy infant. Nanda et al^[11]^ found successful twin pregnancies in a unicornuate uterus with one fetus in the noncommunicating rudimentary horn. Full-term rudimentary horn pregnancies, including in our case, benefit from close and scheduled production inspection.

**Table 1:**
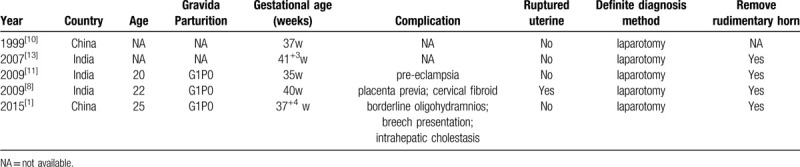
Successful rudimentary horn pregnancy.

When a rudimentary horn with a functional endometrium is diagnosed before pregnancy, it is necessary to excise for fear of menstrual blood retention and obstetrical complications. Abnormal pregnancies can also take place in noncommunicating rudimentary horns due to zygote migration and some other uncertain reasons. For patients diagnosed during pregnancy, immediate surgical management is recommended.^[12]^ The use of methotrexate (MTX) may be helpful during early pregnancy. The degree of development of the rudimentary horn, such as the endometrial function, musculature, and uterine shape and size, is closely related to pregnancy outcome. We can turn to ultrasound and MRI for accurate measurement of the muscle layer thickness. Detailed and scientific labor inspection is important. Patients with rudimentary horn pregnancies with an uncommunicating cervix can only undergo delivery via cesarean section.

## 4 Conclusion

The case of a rudimentary horn pregnancy presented in this report was a rather rare full-term delivery. Rudimentary horn pregnancy is difficult to diagnose and can be found occasionally by surgery. Although rudimentary horn pregnancy is rare, clinicians should pay attention to this life-threatening condition. The degree of development of the rudimentary horn, such as the endometrial function, musculature, and uterine shape and size, is closely related to pregnancy outcome. An increasing number of studies suggest that 3D ultrasound and MRI are helpful for making accurate diagnoses. A rudimentary horn with a functional endometrium should be disposed of by surgery once it is definitely diagnosed. Rudimentary horn pregnancies with a weak muscular layer are suggested to be dealt with as early as possible through MTX or surgery in the case of uterine rupture. Detailed and scientific labor inspection is important.

## 5 Informed consent

Written informed consent was obtained from the patient for publication of this case report and accompanying images.

## 6 Ethical approval

All procedures performed in the study were in accordance with the ethical standards of Medical Ethics Committee of Qilu Hospital of Shandong University and with the 1964 Helsinki declaration and its later amendments or comparable ethical standards. The publication of this case report received consent from the patient.

## Author contributions

**Data curation:** Yu Zhang, Xue Zhang.

**Investigation:** Yu Zhang.

**Writing – original draft:** Yu Zhang.

**Writing – review & editing:** Yu Zhang, Peishu Liu, Yingxin Pang, Zhe Zhao.
